# Magnetized Gold and Tonic Pills

**Published:** 1847-12

**Authors:** 


*Magnetized Gold and Tonic Pills.*—Human ingenuity is equal to the demands of unsatisfied ignorance in the matter of medicine. On looking over the catalogue of nostrums which have been in repute within half a dozen years, together with those now enjoying a full measure of popularity, it would seem almost impossible to edge a new quack article into an already overstocked New England market. But the ever-craving appetite of that great multitude of unreflecting men and women, who are always ready to swallow down impositions under the potent name of medicine, is scarcely satisfied with the variety offered for their acceptance; and it is a lamentable truth, that the patent medicine disease is extending, and the cunning manufacturers of depopulating compounds are glorying in the success of their thousand and one devices to physic the nation. At this juncture of affairs in the system of wholesale humbuggery, one Gridley, of Southampton, Mass., has marched boldly into the arena with a new pill—the mightiest of all. Already, the lame, the halt and the blind, begin to cry for them, as the proprietor says of the worm lozenges—yes, they will sell, because there is magic in the idea of a *golden pill.* Then they are so perfectly safe, too, that box upon box may be taken with impunity, thus prolonging the pleasure indefinitely. Under the schedule of directions we read that "*An adult may take one of the gold pills after breakfast and another after supper, for fifty days.*" What an obvious improvement over the antiquated plan of a dose once or twice a week! In a circular addressed to physicians, one of which is before us, are the following statements: "The base of the gold pill is chloride of gold, iodine and chlorine, exalted nearly one hundred fold by galvanic forces. They are so powerfully magnetic that they generally quiet the nervous system, and in all susceptible persons, produce more or less inclination to sleep, of the most pleasant and refreshing kind. They excite the organs of generation, are a cure of sterility, arouse the system generally, permanently and safely." This quotation explains the abomination that lurks under the guise of a golden pill. Before condemning the British government for winking at the violation of a wholesome, righteous law of China, against the habitual consumption of opium by the people, we had better look into the manner of feeding the ignorant martyrs to imaginary maladies with that seductive drug at home. If morality is not absolutely put to the blush by the insinuation in regard to the specific effects of these "golden pills" on the procreative apparatus, it is enough to make one ashamed of a state of society that tolerates these brazen-faced inroads upon decency.—*Boston Med. & Surg. Jour.*

